# The impact of place perception and environmental behavior in carbon-neutral theme parks on health perception assessment

**DOI:** 10.3389/fpubh.2026.1854343

**Published:** 2026-06-22

**Authors:** Shuang Qiao, Chenghao Fu, Hongxia Xiong, Andy Adcroft

**Affiliations:** 1 College of Education and Art, Shandong Institute of Petroleum and Chemical Technology, Dongying, Shandong, China; 2 Dongbei University of Finance and Economics, Dalian, China; 3 Chengdu Vocational University of the Arts, Chengdu, China; 4 Birkbeck, University of London, London, United Kingdom

**Keywords:** carbon-neutral characteristic perception, carbon-neutral theme park, environmental behavior, health perception, place perception

## Abstract

**Introduction:**

In the context of accelerating urbanization and shifting climate change, this study investigates the interplay between place perception, environmental behavior, and health perception within carbon-neutral theme parks, with a particular focus on the independent contribution of carbon-neutral characteristics beyond general urban green space attributes.

**Methods:**

To achieve this, three carbon-neutral theme parks in Beijing—each possessing a distinct gradient of carbon-neutral features—were selected as a comparative case portfolio. A multi-dimensional data collection approach was adopted, integrating web crawling, questionnaire surveys, computer vision analysis, natural language processing, and LDA topic modeling to develop a theoretical framework linking place perception, pro-environmental behavior, and health perception. Structural equation modeling, along with Bootstrap mediation and moderation tests, was applied to elucidate the influencing pathways and interrelations among these dimensions.

**Results:**

The findings indicate: (1) Landscape visual characteristics, especially green view index, are positively associated with physical comfort. (2) Place emotional cognition is positively correlated with health perception and is also tied to indirect pathways via pro-environmental behavior, with place attachment exhibiting relatively stronger mediated associations with health perception;(3) Carbon-neutral characteristic perception remains independently associated with health perception after controlling for general place perception, varying by park infrastructure. (4) Pro-environmental behavior mediates the place perception–health perception link; ecological experience has the strongest association with psychological restoration.

**Discussion:**

Taken together, this study empirically identifies an independent association between carbon-neutral characteristic cognition and health perception, which is distinct from the effects of general green space. It further reveals how sensation, emotion, behavior, and health are theoretically interconnected within carbon-neutral theme parks, thereby providing theoretical and practical references for differentiated carbon-neutral park design in terms of landscape optimization, behavioral guidance, and health promotion.

## 1 Introduction

The paper investigates how urban green spaces (UGS), particularly urban parks, shape mental and physical well-being. Amidst accelerating urbanization, numerous challenges, such as climate change, ecological degradation, and threats to public health, have emerged as major policy concerns worldwide (1–3). To illustrate, UGS such as urban parks have been proposed as a mitigating factor against air pollution, heat island effects, noise disturbance, and psychological stress, owing to their beneficial influence on physical and mental health and social well-being through the provision of ecosystem services (4). This relationship concerning heat island effects is further refined by An et al. (5), who showed that landscape changes exhibit nonlinear thresholds when regulating urban heat island dynamics, thereby implying that spatial configuration and landscape structure directly shape thermal environments and heat-related conditions in cities. Urban parks facilitate physical activity, reduce mental stress, and enhance social cohesion (6–9). Importantly, these advantages, especially those tied to health, do not depend solely on the mere presence or physical accessibility of green spaces but are more profoundly embedded in the complex psychological and behavioral processes underlying human-environment interaction (10, 11). Prior studies have revealed that the public’s place perception of green spaces together with the pro-environmental behavioral intentions or actual behaviors (especially low-carbon behaviors) stimulated within such environments, collectively constitute a core pathway that shapes individuals’ health perception and experience (12, 13).

Although the linkages between perception, behavior, and health within conventional urban green spaces have been thoroughly explored (14–16), research focusing on parks situated under the specific thematic framework of “carbon neutrality” remains at an early stage of development. Carbon-themed parks pursue both ecological functions and the educational objective of conveying low-carbon concepts while steering public behavior. As innovative environments, they merge ecological low-carbon concepts with recreational uses, thereby directly promoting visitors’ low-carbon actions and improving their health and well-being. Consequently, they are more than mere extensions of traditional urban parks – a notion that can be traced back to earlier ideas of low-carbon ecological corridors linking urban and rural areas (17). While existing literature focuses on low-carbon landscape technologies, carbon sequestration assessment, macro-level management strategies (18, 19), the general health benefits of green space (20, 21) and public willingness to pay for low-carbon policies (22, 23), the user perspective is often absent. From a macro-policy perspective, Ji and Wang (24) demonstrated that the phased expansion of China’s national carbon emissions trading system can facilitate the transition toward carbon neutrality while reducing economic adjustment costs. Their findings underscore the importance of complementary public engagement mechanisms beyond market-based regulation. In this regard, carbon-neutral theme parks may serve as micro-scale behavioral intervention spaces that translate national carbon-neutrality goals into everyday environmental awareness and low-carbon practices (24). This paper addresses that gap by concentrating on user experience and psychological well-being. The remainder of the paper is organized as follows. It will first review the existing literature in order to formulate hypotheses, prior to describing the employing web crawling, questionnaire surveys, and multiple data analysis methods. Subsequently, results are then presented, followed by a discussion of their implications for both theory and practice.

## 2 Literature review and hypotheses

To what extent does our perception of a given space, along with the environmental behaviors it elicits, affect our health? Mohamad and Hussein (25) in their study of urban parks in Kuala Lumpur, observed that natural scenery and engaging landscapes within parks exert considerable restorative effects on residents’ health. Liu et al. (26) taking urban parks in Macau and Fuzhou as research subjects, revealed that the perception of park landscape characteristics positively contributes to restorative perception. That is, the stronger the respondents’ perception of park landscape features, the higher their level of restorative perception. Ramkissoon et al. (27) proposed that when individuals develop a functional reliance on green space environments, this emotional attachment may also generate positive health restoration benefits, including pleasant psychological feelings and comfortable physiological recovery, by evoking positive memories. Accordingly, earlier studies confirm the direct impact of park place perception on health outcomes. Dong et al. (28) further demonstrated that climate-related perceptions, especially perceived heat risks, serve as mediators in the relationship between urban spatial characteristics and physical activity. This finding supports that visitors’ perceptions of environmental quality can serve as a critical psychological mechanism linking place characteristics to health-related behaviors (28). Existing evidence suggest that the public’s visual preferences for parks, assessments of environmental quality, and the psychological responses they evoke are key determinants in their usage frequency, satisfaction, and even overall well-being (29–31).

Arousal theory and self-regulatory attitude theory suggest a link between recreationists’ perception and emotion (32). Perception and evaluation are factors in environmental stimulation and subsequent emotion generation. Bagozzi (33) suggests that emotional arousal influences affective responses, which further influences individuals’ behavioral intentions and attitudes. Place perception often transcends objective physical characteristics, encompassing individuals’ comprehensive cognition and evaluation of park equity, accessibility, safety, quality, and neighborhood environment (34–38), and also significantly influences residents’ life satisfaction and subjective well-being. Some studies have proven that exposure to natural or ecologically appealing environments can enhance individuals’ sense of nature connectedness and environmental values, thereby increasing their willingness to adopt low-carbon or pro-environmental behaviors (e.g., energy-saving consumption, green travel) (12, 39, 40). Currently, little research has examined whether carbon-neutral characteristics provide restorative benefits that extend beyond the effects of conventional urban green space qualities. In summary, this study proposes the following hypotheses regarding carbon-neutral theme parks:

*H1*: The visual characteristics of landscapes within carbon-neutral theme parks exhibit a positive association with visitors’ health perception, given that high-quality visual environments enhance physical comfort and facilitate psychological restoration via sensory stimulation.

*H2*: Within carbon-neutral theme parks, place emotional cognition demonstrates a positive correlation with health perception, as emotional connection reinforces the sense of belonging experience, thereby enhancing environmental satisfaction.

*H3*: In carbon-neutral theme parks, carbon-neutral characteristic perception exerts a significant independent positive association with health perception; this correlation remaining significant after controlling for landscape visual characteristics and place emotional cognition, thus confirming that carbon-neutral-specific mechanisms generate distinct health promotion effects beyond general green space quality.

*H4*: In carbon-neutral theme parks, place perception is positively associated with environmental behavior, whereby high-quality place environments stimulate visitors’ willingness for active exploration and interaction.

Moreover, a growing body of research illustrates how urban parks stimulate and encourage a variety of pro-environmental behaviors, which are in turn closely tied to health benefits. For instance, Dzhambov and Dimitrova have demonstrated that residents’ visits to urban parks are associated with multiple positive health outcomes, including alleviating health anxiety, promoting physiological health recovery, and providing opportunities for social interaction (41, 42). Studies conducted by Zhang and Yang have confirmed that, from a physiological health perspective, parks offer settings for various forms of physical activity such as walking and running, which help lower the risk of chronic diseases (43). From a mental health perspective, exposure to natural environments can effectively facilitate stress recovery, improve mood states, sharpen attention, and elevate subjective well-being (27, 44, 45). Park facilities thus serve as key factors influencing both the frequency of park visitation and the duration of physical activity (46–48). Aligned with this line of reasoning, a study by Dong et al. (49) found that mobility-supportive neighborhood environments can strengthen social relationships through increased social interaction, thereby underscoring how supportive environmental settings generate broader well-being benefits beyond physical health. Research by Zhou et al. (50) suggests that the spatial configuration and accessibility of neighborhood green spaces have been shown to positively shape residents’ recycling behaviors. Korpilo et al. (51) argue that the adoption of low-carbon behaviors, such as actively choosing to walk or cycle to parks, not only directly cuts down carbon emissions but also constitutes, in itself, beneficial physical activity for both body and mind, thereby forming a synergistic “behavior-health-environment” gain cycle. This study proposes the following hypotheses concerning the perception-behavior-health relationship in carbon-neutral theme parks:

*H5*: In carbon-neutral theme parks, pro-environmental behavior is positively associated with health perception, given that behavioral participation fosters mental health benefits through the reinforcement of self-efficacy.

*H6*: Pro-environmental behavior serves as a mediator in the positive relationship between place perception and health perception by enhancing visitors’ psychological engagement and restorative experience.

When examining the role of demographic factors, Wang and Chang (52) revealed that the accessibility of urban parks exerts a substantially stronger impact on the health of older visitors populations compared to younger groups, whereas the vegetation quality of parks significantly enhances the health perception of low-income residents only. Likewise, Angelia et al. (53) found that the magnitude of key psychological factors influencing park usage behaviors, such as nature orientation and social norms, displays systematic differences among groups with differing frequencies of use. These observations suggest that demographic characteristics influence how different populations’ perceive environmental features, their patterns of utilization, and the subsequent health benefits they derived. Accordingly, this study proposes the following hypotheses:

*H7*: In carbon-neutral theme parks, demographic characteristics exert a significant moderating effect on the path from place perception to environmental behavior and ultimately to health perception.

Employing three carbon-neutral theme parks in Beijing as a case study, this study explores how place perception, pro-environmental behavior, and health perception interact, as well as whether the perceived carbon-neutrality attributes of these parks offer restorative benefits on visitors’ perceived health. This investigation seeks to the key factors that amplify such restorative effects, with the objective of informing low-carbon planning strategies within existing urban parks. Drawing upon the theoretical framework and hypotheses outlined above, this study develops a conceptual model that connects place perception, pro-environmental behavior, and health perception within the context of carbon-neutral theme parks. Within this model, landscape visual characteristics, place emotional cognition, and carbon-neutrality attributes constitute the three core dimensions of place perception; pro-environmental behavior is specified as the mediating variable, and health perception serve as the outcome variable. Meanwhile, demographic characteristics function as moderating variables across key pathways. The three characteristic mechanisms of carbon-neutral theme park—carbon cognition activation, behavioral guidance, and self-efficacy enhancement—are embedded within the corresponding pathways of this model, thus furnishing the theoretical foundation for the empirical analysis that follows.

## 3 Data collection and preprocessing

### 3.1 Research object

We selected three representative carbon-neutral theme parks in Beijing as case studies. They form a gradient of carbon-neutral features.

**Figure 1:**
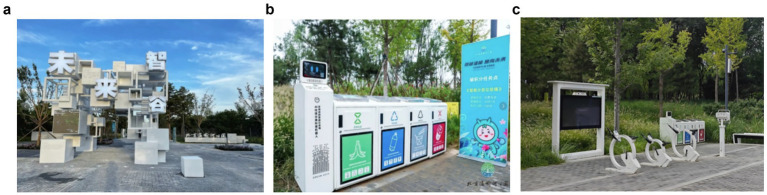
Beijing Wenyuhe Park–Future iValley. **(a)** Entrance landmark. **(b)** Carbon credit waste sorting. **(c)** Carbon credit smart facilities.

Wenyuhe Park–Future iValley (Phase I) has a fully integrated carbon-neutral system. It includes a mature carbon-credit feedback loop that combines renewable energy, ecological carbon sequestration, and behavioral incentive mechanisms (Figure 1).

**Figure 2:**
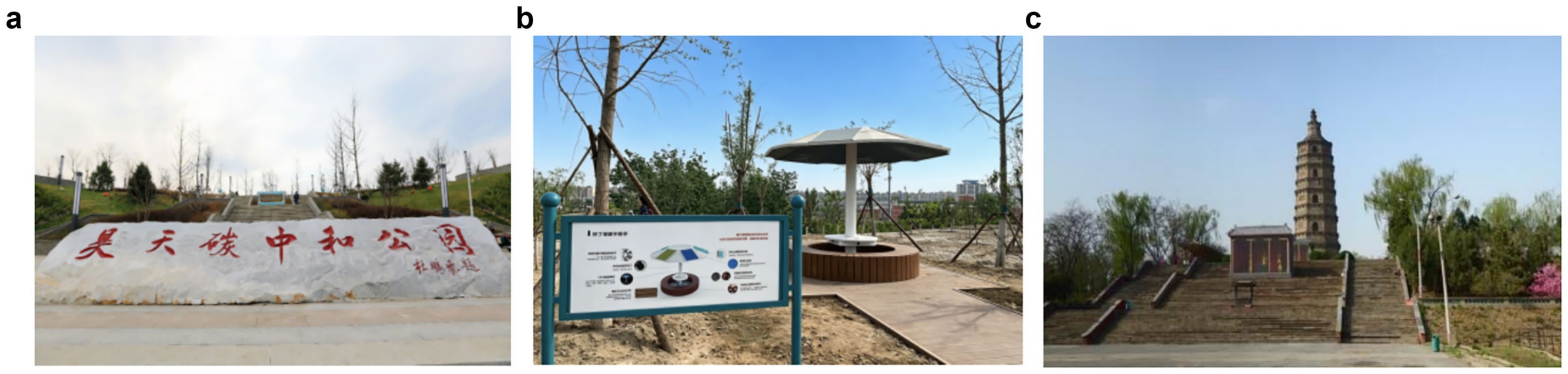
Beijing Haotian Carbon-Neutral Park. **(a)** Entrance landmark. **(b)** Carbon-themed landscape. **(c)** Ecological wetland landscape.

Haotian Carbon-Neutral Park represents a technology-driven carbon reduction model focused on construction-phase carbon control, with limited visitor-facing carbon interaction systems (Figure 2).

**Figure 3:**
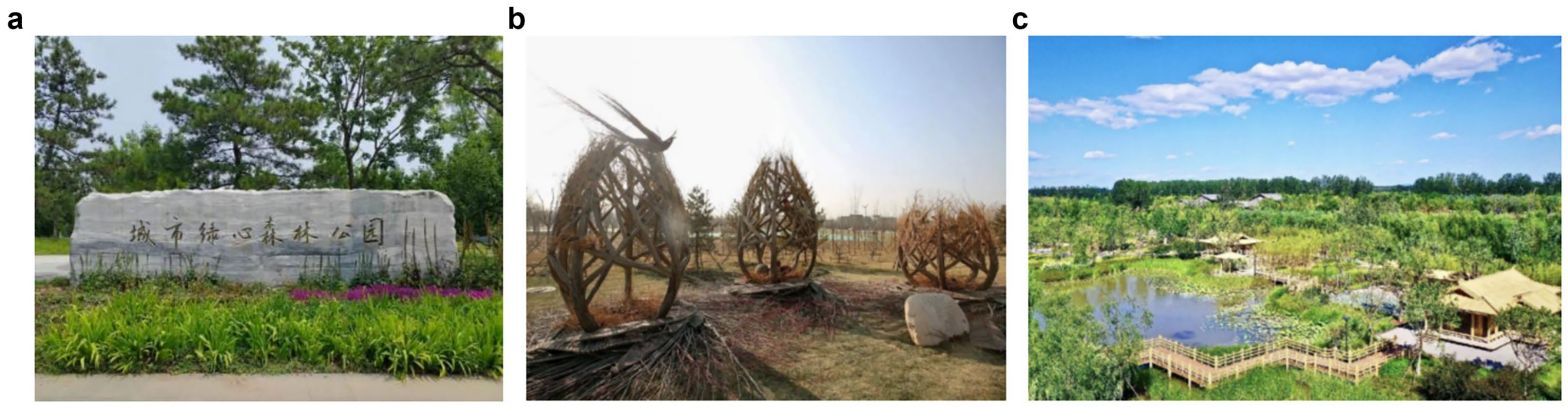
Beijing Urban Green Heart Forest Park. **(a)** Entrance landmark. **(b)** Carbon-themed landscape. **(c)** Ecological wetland landscape.

Urban Green Heart Forest Park represents a large-scale ecological restoration model with partial carbon-neutral experience pathways introduced in recent years, emphasizing carbon sequestration and ecological infrastructure (Figure 3).

These three parks differ in scale, technological approach, and function. Wenyuhe Park contains rich carbon-neutral themed landscapes and low-carbon interactive facilities. It builds a complete closed-loop system spanning construction, renewable energy, ecological sinks, and visitor behavior guidance. In contrast, Urban Green Heart Park’s carbon-specific features center on renewable energy and carbon sink offsetting. Haotian Park’s carbon-neutral characteristics are mainly construction-phase low-carbon technologies. Both have far fewer carbon-themed landscape elements than Wenyuhe Park. This variation provides a strong empirical basis for studying how place quality influences visitor health in carbon-neutral theme parks.

### 3.2 Data collection methods

We collected data using two approaches: online data collection and questionnaire surveys. Online data comprised user-generated content from Dianping, Xiaohongshu, Ctrip, and Weibo, including text comments, images, and ratings. Questionnaire surveys were conducted primarily through online distribution via the Wenjuanxing platform, supplemented by a small number of offline questionnaires intercepted at the main entrances and exits of each park. The online-first approach enabled broader geographic coverage of Beijing residents who had visited the three parks, reducing seasonal and weather-related sampling bias inherent to purely on-site data collection. We obtained 420 valid responses, providing data on visitors’ place perception, environmental behavior, and self-reported health perception.

#### 3.2.1 Implementation of web scraping technology

Online data collection lasted from April 2022 to December 2024, covering 32 months of park operation. This period includes a complete annual cycle, enabling the research to account for the impact of seasonal and weather changes on visitors’ emotions and health perceptions. The extended data collection period enhanced the diversity of visitor feedback and improved sample validity, providing a solid temporal foundation for studying the relationship between environmental awareness and health outcomes (Table 1).

**Table 1:** Statistics of multi-platform UGC data collection for three parks.

Data platform	Data type	Wenyu River Park	Green Heart Park	Haotian Park	Total	Time span	Valid samples
Dianping	Text reviews, ratings	178 entries	164 entries	156 entries	498 entries	2022.04–2024.12	452 entries
Xiaohongshu	Image-text notes, images	102 posts	95 posts	89 posts	286 posts	2022.04–2024.12	258 posts
Ctrip Travel	Travel blogs, reviews	73 entries	68 entries	64 entries	205 entries	2022.04–2024.12	187 entries
Weibo	Topic discussions, images	54 entries	51 entries	47 entries	152 entries	2022.04–2024.12	138 entries
Total	–	407 entries	378 entries	356 entries	1,141 entries	–	1,035 entries

In order to utilize this data, scripts were written in Python to collect data, used the Requests library to crawl web pages, and used BeautifulSoup and Scrapy frameworks to parse HTML structures and extract the necessary data. On the Dianping platform, methods were used to bypass the anti-crawler system, such as simulating user login, setting a random access interval of 3–8 s, and rotating User-Agent strings so that user comments, ratings, and timestamps can be collected. These are key data. For Xiaohongshu, note titles, full text, image links and user interaction data through its mobile API interface were used. For Ctrip and Weibo, keywords such as “Wenyu River Low-Carbon Park”, “Haotian Carbon-Neutral Park”, “Urban Green Heart Forest Park,” and “Carbon Neutral Park Beijing” were used to search, and then systematically downloaded relevant travel posts and discussion content.

#### 3.2.2 Questionnaire scale design and distribution

We designed the questionnaire based on established environmental psychology and behavior scales. Computational analyses of user-generated content, including sentiment analysis and topic modeling, were used only as supplementary references to help contextualize park-specific environmental themes and visitor experiences. The place perception dimension references Williams’ Place Attachment Scale (54) and Kaplan’s Attention Restoration Theory (55); the environmental behavior dimension is grounded in Stern’s Environmental Behavior Theory (56) with adaptive adjustments for the park context; the health perception dimension integrates the WHO-5 Well-Being Scale (57) and the Restorative Environment Scale (58). Building upon this theoretical framework and in light of the spatial specificity of carbon-neutral theme parks, this study adds “carbon-neutral characteristic perception” as an independent measurement sub-dimension under the place perception dimension, comprising four items: perceived visibility of low-carbon facilities, perceived immediate value of the Carbon Points system, cognitive impact of carbon sink plant displays, and perceived differentiation of the low-carbon theme. This sub-dimension is positioned alongside landscape visual characteristics and place emotional cognition within the structural equation model, enabling independent identification of the net contribution of carbon-neutral-specific effects on health perception while controlling for general place perception variables, thereby achieving effective methodological separation of carbon-neutral-specific effects from the general effects of ordinary urban green spaces.

The final questionnaire has 28 main measurement items and 5 demographic items (33 items total, Table 2). All items use a five-point Likert scale (1 = strongly disagree, 5 = strongly agree). Data were collected primarily through online distribution via the Wenjuanxing platform, targeting Beijing residents who had visited at least one of the three carbon-neutral theme parks, supplemented by a small number of offline questionnaires intercepted at the main entrances and exits of each park on both weekdays and weekends. The online-first approach was adopted to ensure broader geographic coverage and to reduce seasonal sampling bias associated with purely on-site data collection. A total of 500 questionnaires were distributed, of which 463 were collected; after removing invalid responses — including questionnaires with missing values, straight-line responses, and completion times below 180 s — 420 valid samples were retained. The overall Cronbach’s *α* value of the scale is 0.921, the KMO value is 0.914, and Bartlett’s test of sphericity yielded a *χ*^2^ value of 2,156.84 (*p* < 0.001), with all item factor loadings exceeding 0.6, demonstrating good internal consistency and structural validity.

**Table 2:** Questionnaire scale structure and measurement indicator system.

Measurement dimension	Sub-dimension	Number of items	Measurement indicator examples
Place perception	Landscape Visual Characteristics	5	Green view index perception, color harmony, spatial openness
	Place Emotional Cognition	5	Place identity, place attachment
	Carbon-neutral Characteristic Perception	4	Perceived visibility of low-carbon facilities, perceived immediate value of Carbon Points, cognitive impact of carbon sink plant displays, perceived differentiation of low-carbon theme
Environmental behavior	Low-carbon Behavior	4	Green travel, waste sorting, use of new energy facilities, experience of the “Carbon Points” Park Visiting System
	Ecological Experience	3	Nature observation, environmental education participation, ecological interaction
Health perception	Physical Comfort	3	Air quality, temperature and humidity, noise perception
	Psychological Restorativeness	4	Stress relief, attention restoration, emotional improvement, sense of vitality
Personal information	Demographic Characteristics	5	Gender, age, education, occupation, monthly income, visit frequency
Total		33	

#### 3.2.3 Synchronous collection of text and image data

**Figure 4:**
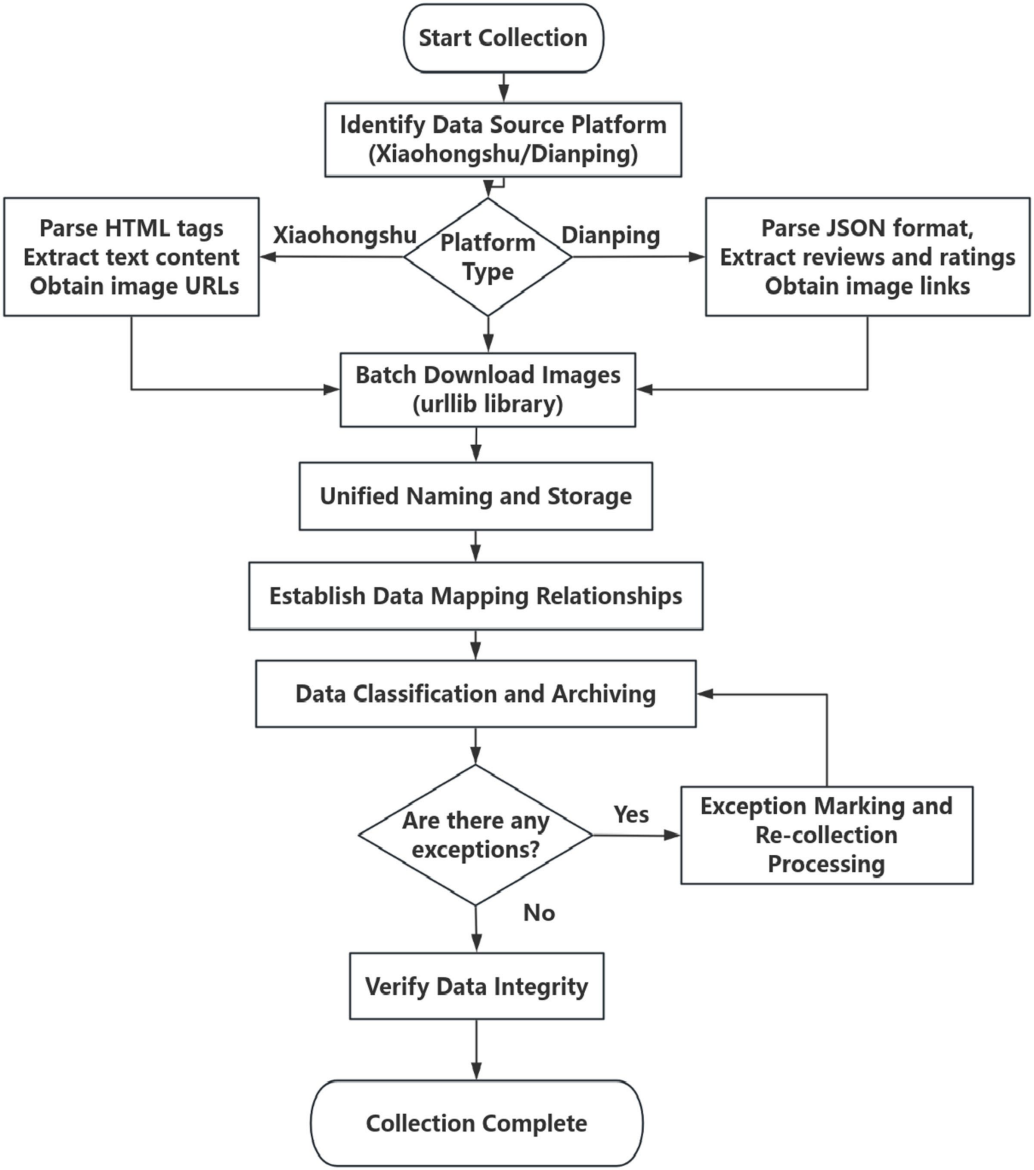
Schematic diagram of synchronous text and image data collection process.

The synchronous collection of text and image data relies on a timestamp matching mechanism to achieve multimodal data association. For image-text notes on the Xiaohongshu platform, the web scraper extracts image URLs by parsing <img> tags in HTML while obtaining text content, then batch downloads image files using the urllib library and stores them with unified naming following the rule of “platform name_user ID_posting time”. Comment data from the Dianping platform is parsed in JSON format, from which comment text, ratings, image links and other information are extracted, and unique identifiers are established to construct one-to-many mapping relationships between text and images. Pictures are sorted and stored according to upload date and category (such as landscapes, infrastructure, events), while text data remains the same, and all related metadata is also preserved (Figure 4).

### 3.3 Data preprocessing

Data preprocessing ensures the accuracy of data through a complete quality inspection framework, which is essential for subsequent analysis.

First, text was cleaned up and standardized using regular expressions to remove HTML tags, websites, special symbols and emoticons. The jieba tool was used to do word segmentation and to compile a list of stopped words which filtered out useless words. Those samples that were too short (less than 10 words) or too long (more than 500 words) were rejected. The TF-IDF method was used to extract the main feature words and generate a standardized text feature vector matrix. The processed data can be used for emotion classification and theme analysis.

**Figure 5:**
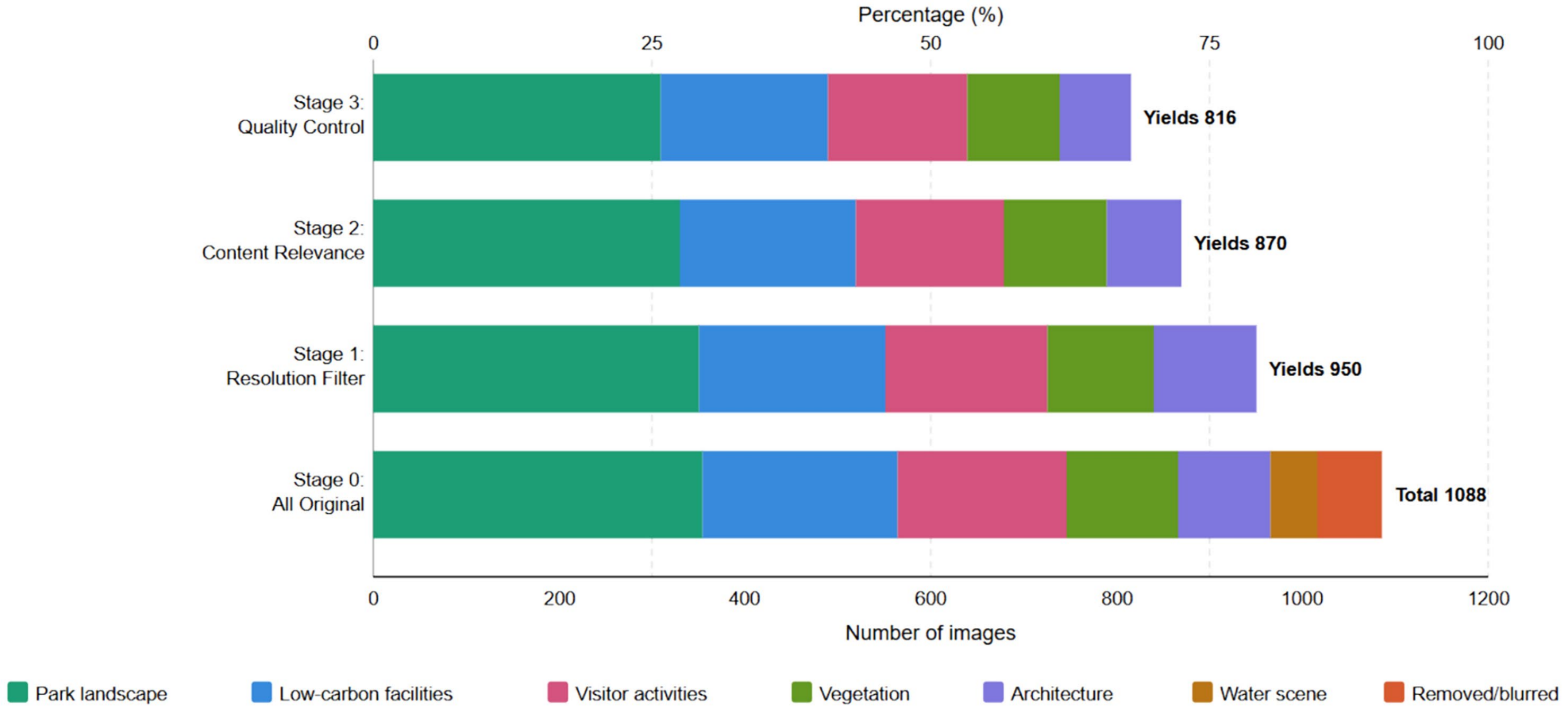
Image data quality screening process and results distribution.

Second, a three-step method was used to evaluate picture quality (Figure 5). The first step was technical inspection, using the PIL library to confirm the resolution of the picture, and deleting all the photos that are less than 800×600 pixels. The next step was content screening. A convolutional neural network called ResNet-50 was used to classify scenes and only keep photos of parks, ecological facilities or tourists’ activities. Finally, the integrity of the scene was checked to see if the composition is good and the information is enough. After these three rounds of screening, 816 valid images remained from the original 1,088, yielding a retention rate of 75%.

Third, the reliability and validity of the questionnaire data was assessed. Cronbach’s *α* was used to measure internal consistency, and the *α* value of the whole scale was 0.921. The KMO value is 0.914, and Bartlett’s test of sphericity yielded a *χ*^2^ value of 2,156.84 (*p* < 0.001), and the factor load of all items exceeds 0.6, which shows that the scale has strong structural validity.

Fourth, multi-source data integration was conducted. A three-tier table structure was constructed using a MySQL relational database, with records across different tables linked via anonymized temporal and spatial identifiers. Text sentiment scores, landscape image indicators, and questionnaire responses were standardized to a 0–1 range and integrated into a unified data matrix comprising 1,035 user-generated content entries, 816 valid images, and 420 questionnaire samples.

The UGC text/image data and questionnaire responses are from different populations, so individual-level matching is not possible. This study therefore adopts a stratified integration strategy: AI-derived indicators (e.g., BERT sentiment, SegNet green view, LDA topic weights) are extracted from the full UGC/image datasets and reflect aggregate-level (park-wide) characteristics. For each park, we aggregated AI indicators as the mean value across all valid samples from that park (e.g., mean BERT sentiment score, mean green view index). We then standardized all aggregated AI indicators to a 0–1 range based on the full sample distribution across the three parks. This procedure avoids cross-park bias because the same global distribution is applied to all parks. Questionnaire responses provide individual-level perceptual and behavioral measures. After standardization, the aggregate-level AI indicators are incorporated into the SEM as auxiliary observed indicators for corresponding latent variables (e.g., LDA weights for “environmental behavior”), alongside individual-level questionnaire items.

This achieves multi-source integration at the construct level (jointly specifying latent variables), not at the individual sample level. The AI indicators serve as objective, park-wide complements to subjective person-specific measures, avoiding pseudo-individual-level data fusion.

### 3.4 Research framework

**Figure 6:**
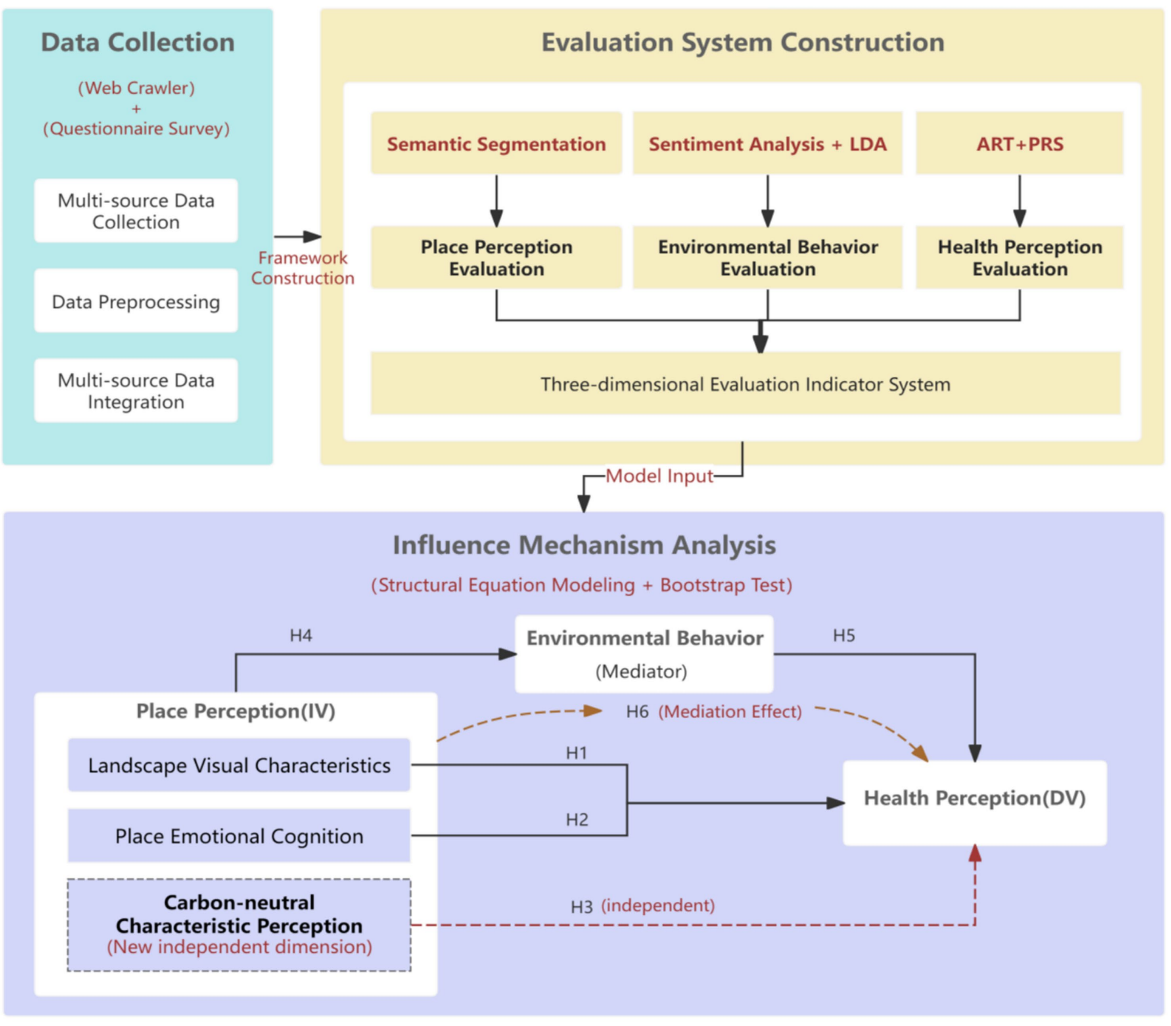
Research technical framework diagram.

We organized the analysis into three sequential stages: data collection, indicator construction, and mechanism analysis (Figure 6).

Data collection. We integrated user-generated text from Dianping, Xiaohongshu, Ctrip, and Weibo with park images and questionnaire data from Wenjuanxing. All data underwent standardized preprocessing. Images passed a structured three-stage screening. The questionnaire measured place perception, environmental behavior, and health perception.

Indicator construction. Landscape visual characteristics were quantified using computer vision, place emotional cognition was identified using natural language processing (BERT), and environmental behavior themes were classified through LDA topic modeling. We then built a unified health perception composite index. This allowed us to integrate multi-source data within a single analytical framework.

Mechanism analysis. We used structural equation modeling (SEM) to examine pathways among place perception, environmental behavior, and health outcomes. Bootstrap tests assessed mediation and moderation effects. Carbon-neutral characteristic perception entered the model as an independent latent variable, enabling us to estimate its net contribution to health perception after controlling for general place perception variables.

Integration occurred at the construct level rather than the individual sample level. Specifically, aggregate AI-derived indicators and individual questionnaire items jointly specified latent variables in the SEM. This stratified integration strategy enabled the combination of multi-source data while avoiding direct matching between AI-derived indicators and individual questionnaire responses. Further details are provided in Section 4.4.1.

## 4 Evaluation methods

### 4.1 Place perception evaluation

#### 4.1.1 Landscape visual characteristics quantification (green view index, color harmony, spatial openness)

Landscape visual characteristics quantification used computer vision technology to perform automated analysis on 816 valid images across the three carbon-neutral theme parks.

Green view index was calculated through the SegNet semantic segmentation algorithm to identify vegetation pixels, with calculation Equation 1 as:

$$\mathrm{GVI}=\frac{P_{\text{green}}}{P_{\text{total}}}\times 100\%$$ (1)

Where GVI represents the Green View Index;

*P*_green_ represents the number of pixels identified as vegetation category in the image;

*P*_total_ represents the total number of pixels in the image.

Color harmony was calculated from hue distribution balance in HSV color space, and spatial openness was evaluated using sky proportion and visual depth. We batch-processed images using the Python OpenCV library and deep learning models. Quantification results show that the three parks’ overall average green view index is 0.56, average color harmony 0.70, and average spatial openness 0.62, with notable differences among parks (Table 3). Green Heart Park had the highest GVI (0.61), followed by Wenyu River Park (0.58) and Haotian Park (0.50). Color harmony followed a similar pattern. Spatial openness was highest in Wenyu River and Haotian parks. Overall, the landscape visual quality of the three parks is at a good level, providing a solid visual environment foundation for the positive association between place perception and health perception.

**Table 3:** Landscape visual characteristics quantification indicator system and calculation methods.

Indicator type	Calculation method	Value range	Wenyu River Park	Green Heart Park	Haotian Park	Overall mean	Standard deviation	Technical tools
Green View Index	Vegetation pixels/Total image pixels	0–1	0.60	0.61	0.50	0.57	0.14	SegNet Semantic Segmentation
Color Harmony	1 – Hue variance/Maximum hue variance	0–1	0.73	0.70	0.66	0.70	0.10	HSV Color Space Analysis
Spatial Openness	(Sky proportion × 0.6) + (Visual depth index × 0.4)	0–1	0.59	0.64	0.63	0.62	0.12	Edge Detection + Perspective Geometric Analysis

The green view index obtained from SegNet semantic segmentation and the scene classification results from ResNet-50, after standardization, were employed as objective observed indicators for the “landscape visual characteristics” latent variable in the structural equation model. Together with questionnaire items on green view perception, color harmony, and spatial openness, these indicators constitute the measurement system for this latent variable, achieving multi-source complementarity between objective image data and subjective questionnaire data.

#### 4.1.2 Place emotional cognition recognition and measurement

We used natural language processing to analyze text data, focusing on emotion and semantics. In the emotional analysis, we use BERT pre-training model to evaluate the emotional tendency of 1,035 effective samples, classify them into three categories: positive, neutral or negative, and give them corresponding emotional scores. Through transfer learning, with the help of SnowNLP Emotional Dictionary and THUCNews Corpus, the accuracy of the model on the test set reached 86.7%. The data show that positive sentiment accounts for 67.8%, neutral sentiment 24.6%, and negative sentiment only 7.6%. The sentiment orientation distribution is similar across the three parks (Wenyu River Park 68.3%, Green Heart Park 69.1%, and Haotian Park 65.9% for positive sentiment), which shows that the tourists’ evaluation of the park environment is generally good.

**Figure 7:**
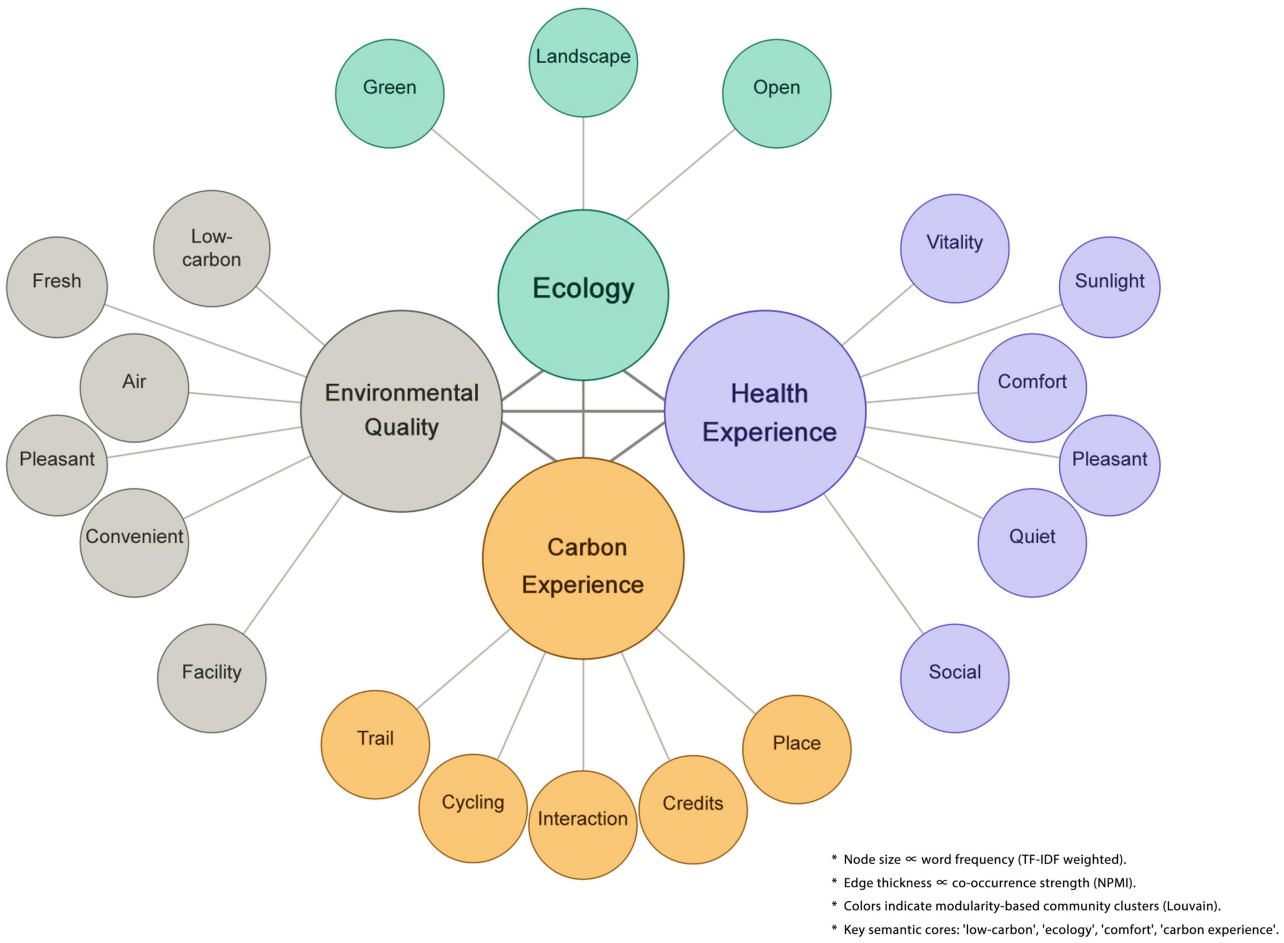
Semantic network diagram of high-frequency feature words for place perception. Node size is proportional to word frequency; edge thickness represents the strength of co-occurrence (normalized mutual information).

To further verify the content validity of the observed indicators for the two latent variables of place emotional cognition and carbon-neutral characteristic perception, this study employs co-word analysis to construct a semantic network. High-frequency feature words are extracted through jieba word segmentation to build a word co-occurrence matrix quantifying semantic association strength. The top 50 feature words by frequency are screened and the Gephi tool is utilized to generate a semantic network map (Figure 7). The results indicate that words such as “Ecology,” “Environmental Quality,” “Health Experience,” and “Carbon Experience” constitute core nodes: among these, “Low-carbon” and “Carbon Credit Experience” form direct semantic correspondence with observed indicators in the carbon-neutral characteristic perception sub-dimension, thereby corroborating the content validity of this sub-dimension’s item design; meanwhile, words such as “Ecology” and “Comfort” are highly consistent with the semantic orientation of place attachment and place identity items within the place emotional cognition dimension, providing semantic support from user-generated content for the validity of the emotional cognition dimension’s measurement items.

We then used the five place emotional cognition items from the questionnaire to quantitatively assess place identity and attachment. Place emotional items were designed (e.g., “I believe this park aligns with my environmental protection philosophy,” “The environmental quality of this park makes me feel proud,” etc.) to complete data collection. The measurement method combined descriptive statistics and exploratory factor analysis. Software was employed to calculate the mean and standard deviation of each item to assess the actual intensity of place identity and place attachment. Factor analysis was used to verify the validity of the two-dimensional structure by selecting factors with eigenvalues greater than 1 and examining the KMO value and the cumulative variance explained. Pearson correlation coefficient analysis to study the relationship between place identity and attachment, which provides a quantitative method and analytical basis for exploring how place perception affects environmental actions and health perceptions in the future. In order to understand the theme framework and conceptual relationship in tourists’ comments more clearly, we also use co-word analysis to generate a semantic network based on sentiment analysis, which graphically shows the main concerns of tourists about environment and health-related experiences.

The sentiment analysis results served as supplementary evidence to interpret visitors’ emotional responses and to check consistency with questionnaire-based measures. Together with questionnaire items on place identity and place attachment, these indicators form a complementary integration of objective and subjective data, jointly characterizing visitors’ emotional evaluation dimension of the park environment.

### 4.2 Environmental behavior evaluation

#### 4.2.1 Behavior theme extraction and classification

This study employs the LDA (Latent Dirichlet Allocation) topic model to conduct unsupervised learning on 1,035 valid texts from three carbon-neutral theme parks. By combining the topic-word distribution matrix with artificial semantic analysis, five categories of environmental behavior themes are identified (Table 4).

**Table 4:** Environmental behavior theme classification system and feature word distribution.

Behavior category	Behavior description	High-frequency feature words	Topic weight	Text proportion
Low-carbon Behavior	Green travel, Waste sorting, “Carbon Credit” Experience	Cycling, Walking, Sorting, Charging station, New energy, Carbon Points	0.27	30.8%
Ecological Experience	Nature observation, Organism identification, Sensory perception	Plants, Birds, Seasons, Flowers, Insects, Travel	0.24	27.6%
Pro-environmental Practice	Volunteer activities, Environmental education interactive activities	Volunteer, Publicity, Science education, Interactive, Planting trees	0.18	17.9%
Leisure Recreation	Walking, Exercise, Photography, Social gathering	Walking, Running, Photography, Playing cards, Parent–child, Gathering	0.22	16.5%
Educational Learning	Understanding carbon neutrality technology, Visiting exhibition facilities	Photovoltaic, Carbon neutrality, Exhibition, VR, Technology, Knowledge	0.09	7.2%

Low-carbon behavior accounts for the largest proportion, with a text proportion of 30.8%, which is particularly prominent in Wenyu River Park, directly attributable to its complete carbon credit closed-loop system. Ecological experience accounts for 27.6%, with a relatively higher proportion in Urban Green Heart Forest Park, highly consistent with its abundant ecological resources. Pro-environmental practice and leisure recreation account for 17.9 and 16.5% respectively, while educational learning accounts for 7.2%, primarily concentrated in Wenyu River Park and Urban Green Heart Forest Park.

The distribution reflects park differences: parks with stronger carbon-neutral characteristics showed higher proportions of low-carbon behavior and educational learning; parks with richer ecological resources had more ecological experience; Haotian Park (less perceptible carbon-neutral features) showed more leisure recreation. After standardization, the five-category theme weight distribution serves as an auxiliary observed indicator for the “environmental behavior” latent variable in the structural equation model, jointly achieving multi-source integration of text mining data and questionnaire survey data together with questionnaire items.

#### 4.2.2 Quantification of low-carbon behavior, ecological experience, and pro-environmental practices

Based on Stern’s (56) VBN theory, we designed 10 items measuring three types of environmental behaviors using a 5-point Likert scale. Sub-dimension scores were calculated as item means, creating a continuous indicator of behavioral intensity (Table 5).

**Table 5:** Pro-environmental behavior quantification measurement indicator system.

Behavior Sub-dimension	Number of items	Measurement item examples	Assignment standard	Calculation method
Low-carbon Behavior	4	Choose public transportation to travel to the park	1 = Never, 5 = Always	Mean of 4 item scores
		Consciously sort waste		
		Use new energy sources within the park (charging stations, etc.)		
		Experience the “Carbon Credit” Park Visiting System		
Ecological Experience	3	Observe the carbon sink plants in the park	1 = Never, 5 = Always	Mean of 3 item scores
		Participate in nature education activities (Low-Carbon Science Education Theme Park)		
		Experience ecological changes in different seasons		
Pro-environmental Practice	3	Understand carbon neutrality and related knowledge and technologies	1 = Completely unaware, 5 = Very well aware、1 = Never participate, 5 = Frequently participate、1 = Never, 5 = Always	Weighted average plus bonus (Awareness×0.4 + Participation×0.3 + Recommendation×0.3)
		Participate in environmental volunteer service or publicity activities		
		Recommend low-carbon environmental protection concepts to others		

### 4.3 Health perception evaluation

Health perception evaluation was based on 7 items in the health perception dimension of the questionnaire scale, subdivided into three sub-dimensions for comprehensive assessment.

(1) Physical Comfort assessment used 3 items to measure visitors’ perceptions of air quality, temperature and humidity suitability, and noise levels, with the scale designed referring to ISO 7730 thermal comfort standards and WHO environmental noise guidelines.

(2) Psychological restorativeness assessment used 4 items, based on Kaplan’s Attention Restoration Theory (ART) and the Perceived Restorativeness Scale (PRS), measuring the degree of stress relief, attention restoration effects, emotional improvement status, and vitality enhancement, with items such as “In the park I feel stress is released” and “After leaving the park I feel energized.”

(3) Environmental satisfaction assessment integrated the previous two dimensions, conducting overall evaluation through the comprehensive rating item “I am satisfied with the overall environment of the park.”

### 4.4 Impact mechanism model

#### 4.4.1 Structural equation model construction

The UGC/image data (1,035 entries, 816 images) and questionnaire data (420 responses) come from different populations and are not matched at the individual level. Therefore, this study adopts a stratified integration strategy rather than individual-level merging. AI techniques (BERT, SegNet, and LDA) extract aggregate-level indicators that characterize park-wide features: per-park sentiment distribution, average green view index, and behavioral theme weights. These aggregate indicators are standardized (0–1) and used as objective observed indicators for latent variables in the SEM, alongside individual-level subjective items from the questionnaire. In other words, AI-derived and questionnaire indicators complement each other at the construct (latent variable) measurement level, not at the individual sample level. The independent contribution of AI indicators is demonstrated in Section 5.1.3 (Table 6).

**Table 6:** Path coefficients and effect decomposition of place perception dimensions on health perception.

Place perception dimension	Standardized path coefficient β	Contribution rate	Direct effect	Indirect effect	Total effect	Primary associated sub-dimension
Landscape Visual Characteristics	0.486***	29.3%	0.486	–	0.486	Physical Comfort
Place Emotional Cognition	0.437***	26.3%	0.298	0.139	0.437	Psychological Restorativeness
Carbon-neutral Characteristic Perception	0.312***	18.8%	0.312	–	0.312	Health Perception Composite Index
Total Effect	–	–	1.096	0.139	1.235	–
R^2^ = 0.617	–	–	Direct Effect 68.4%	Indirect Effect 31.6%	–	–
AI Objective Indicators	–	28.4%	–	–	–	Variance Explanation Proportion
Subjective Questionnaire Indicators	–	33.3%	–	–	–	Variance Explanation Proportion

***p < 0.001; indirect effects are generated through the environmental behavior mediation pathway; contribution rates represent the independent contribution proportion of each dimension to the variance explanation of health perception.

The SEM (AMOS 24.0) includes four exogenous latent variables — landscape visual characteristics (LVC), place emotional cognition (PEC), carbon-neutral characteristic perception (CNCP), and environmental behavior (EB) — and one endogenous latent variable: health perception (HP). LVC and PEC represent general place perception dimensions common to ordinary green spaces; CNCP captures visitors’ cognition of low-carbon thematic features (visibility of low-carbon facilities, carbon credit system value, carbon sink plant displays, low-carbon theme differentiation). This specification enables statistical separation of carbon-neutral-specific effects from general green space effects. CNCP’s path coefficient on HP is estimated while controlling for LVC and PEC; significance indicates an independent net contribution beyond ordinary park quality. The three case parks provide a natural gradient in carbon-neutral characteristics (Wenyu River: full carbon credit system; Green Heart: experience route; Haotian: construction-phase technologies), which strengthens the discriminability of CNCP effects.

The composition of observed indicators for each latent variable is as follows. The observed indicators for LVC consist of subjective questionnaire items (green view index perception, color harmony, and spatial openness) and objective image analysis indicators (green view index calculated by SegNet semantic segmentation and ResNet-50 scene classification results). The observed indicators for PEC consist of subjective questionnaire items (place identity and place attachment) and BERT sentiment analysis scores. The observed indicators for CNCP consist of the four newly added subjective questionnaire items and objective indicators derived from the standardized topic weights of the “educational learning” and “low-carbon behavior” categories in the LDA topic model. The observed indicators for environmental behavior consist of questionnaire items on low-carbon behavior, ecological experience, and pro-environmental practice, together with the five-category behavioral topic weight distribution from LDA topic modeling.

The basic Equation 2 of the structural model is:

η=Bη+Γξ+ζ (2)

Where *η* represents the endogenous latent variable vector (health perception); *ξ* represents the exogenous latent variable vector (place perception and environmental behavior); *Γ* represents the path coefficient matrix of exogenous latent variables on endogenous latent variables; and *ζ* represents the residual term.

Model fit was evaluated using multiple goodness-of-fit indices, including *χ*^2^/df < 3, Comparative Fit Index (CFI) > 0.90, Tucker–Lewis Index (TLI) > 0.90, Root Mean Square Error of Approximation (RMSEA) < 0.08, and Standardized Root Mean Square Residual (SRMR) < 0.08.

#### 4.4.2 Path relationships and hypothesis testing

To test H1–H6, we estimated path coefficients using maximum likelihood (ML) and assessed their significance via bootstrapping (2,000 resamples). A path was considered significant when |C. R.| > 1.96 and *p* < 0.05. Modification indices (MI) above 3.84 were inspected for potential model improvements.

For H3 (independent effect of carbon-neutral characteristic perception on health perception), we examined whether its path coefficient remained significant (*p* < 0.05) after controlling for landscape visual characteristics and place emotional cognition. Significance under these controls would indicate that carbon-neutral characteristic perception contributes independently to health perception, beyond general green space quality – supporting H3. To strengthen identification, we exploited the natural gradient of carbon-neutral features across the three parks. Significance was judged by whether the bias-corrected 95% confidence interval excluded zero, following the same bootstrap procedure used for H1–H6.

#### 4.4.3 Mediation effect and moderation effect analysis

We tested the mediating role of pro-environmental behavior using bootstrapping within the SEM framework. The total effect of place perception on health perception was decomposed into direct and indirect effects, and we calculated the mediation ratio to quantify pathway strength. These SEM results reflect associations consistent with our theoretical model and do not imply causation.

For moderation, we examined how demographic characteristics – age, education level, visit frequency, gender, and monthly income – influenced the path relationships. We tested moderation by adding interaction terms in regression analyses, with bootstrapping (2,000 resamples) to assess significance. Simple slope analysis and visualization charts were generated for different moderation levels. We applied the Johnson-Neyman technique to identify the specific range where the moderation effect reached statistical significance.

We also compared groups based on use of the carbon credit system. Visitors who had experienced the Carbon Credit Park Visiting System formed the usage group (*n* = 213); the remaining visitors formed the non-usage group (*n* = 189). Given the gradient distribution of carbon credit availability, most usage-group visitors came from Wenyu River Park, supplemented by relevant visitors from Urban Green Heart Forest Park. We used multi-group SEM to estimate path coefficients separately for the two groups and tested inter-group differences. This allowed us to verify whether the carbon-neutral mechanism independently enhances health perception beyond general green space benefits.

## 5 Research results

### 5.1 Impact of place perception on health perception

#### 5.1.1 Fundamental role of landscape visual characteristics

Path analysis showed that landscape visual characteristics correlated significantly with health perception across all three parks. Among the three visual indicators, the green view index had the strongest positive association with physical comfort. Higher vegetation coverage linked to better perceived air quality and thermal comfort, possibly through microclimate regulation and particle reduction.

Color harmony was mainly associated with psychological restorativeness. Balanced warm and cool tones reduce visual fatigue and promote emotional calm via sensory regulation. Spatial openness corresponded with lower stress levels: panoramic sky views and multi-level landscape depth create an “escape” experience, consistent with attention restoration theory and visual permeability.

The pathways from landscape visual characteristics to health perception followed common patterns across the three parks, but effect intensities varied systematically with differences in green view index, color harmony, and spatial layout (Table 7). Wenyu River Park, which has a high green view index (0.60) and the richest carbon-neutral themed landscape elements, showed the strongest total effect on health perception (0.997). Urban Green Heart Forest Park, with the highest green view index (0.61) and relatively high spatial openness (0.64), achieved a complementary balance between the two indicator types, yielding a total effect of 0.972. Haotian Park, despite comparatively high spatial openness (0.63), recorded the lowest green view index (0.50) and consequently the weakest total effect (0.918). The pattern of path coefficients across the three parks closely mirrored the measured values of green view index, color harmony, and spatial openness, supporting the predictive validity of the quantified landscape visual indicators for health perception pathways.

**Table 7:** Effects of landscape visual characteristics on health perception.

Park name	Green view index Mean	Color harmony mean	Spatial openness mean	Green view index → Health perception path coefficient	Color harmony → Health perception path coefficient	Spatial openness → Health perception path coefficient	Total effect of landscape visual features
Wenyu River Park	0.60	0.73	0.59	0.438***	0.305**	0.254*	0.997***
Green Heart Park	0.61	0.70	0.64	0.412***	0.289**	0.271**	0.972***
Haotian Park	0.50	0.66	0.63	0.356**	0.273*	0.289**	0.918***
Overall Mean	0.56	0.70	0.62	0.402***	0.289**	0.271**	0.962***

***P < 0.001, **P < 0.01, *P < 0.05.

#### 5.1.2 Health effects of emotional cognition

Place emotional cognition, encompassing place identity and place attachment, demonstrates a strong connection with visitors’ self-rated health perception through two parallel associations: a direct pathway associated with higher psychological restorativeness, and an indirect pathway is associated with environmental behavior participation, which is further associated with health perception. Path analysis confirms the transmission mechanism of “emotional response — behavioral participation — health associations between variables.”

The three parks exhibit meaningful differences in place emotional characteristics corresponding to their respective features (Table 8). Wenyu River Park records the highest place identity score (3.94) due to its distinctive carbon credit system, yet the lowest place attachment score (3.52), as its newer themed landscape has not yet cultivated deep emotional bonds. Although its direct effects are the strongest, the high direct effect proportions reduce the relative share of indirect effects, resulting in the lowest mediation proportion (31.8%) despite its highest behavioral participation rates. Urban Green Heart Forest Park records the highest place attachment score (3.89), driven by its abundant ecological resources, generating the highest indirect effect (0.368), total effect (1.071), and mediation proportion (34.4%). Haotian Park’s moderate scores on both dimensions produce intermediate path coefficients and a balanced mediation proportion of 33.5%.

**Table 8:** Comparison of effects of place identity and attachment on health perception.

Park name	Place identity score	Place attachment score	Identity → Health perception direct effect	Attachment → Health perception direct effect	Emotional cognition → Behavior → Health perception indirect effect	Total effect of emotional cognition	Mediation effect proportion
Wenyu River Park	3.94	3.52	0.392***	0.295**	0.321**	1.008***	31.8%
Green Heart Park	3.76	3.89	0.347***	0.356***	0.368***	1.071***	34.4%
Haotian Park	3.82	3.64	0.364***	0.318**	0.343**	1.025***	33.5%
Overall Mean	3.84	3.68	0.368***	0.323***	0.344***	1.035***	33.2%

***p < 0.001, **p < 0.01, *p < 0.05.

The overall mean mediation proportion of 33.2% confirms that environmental behavior consistently serves as a meaningful transmission mechanism across all three parks, with parks characterized by stronger place attachment generating proportionally greater health benefits through behavioral mediation.

#### 5.1.3 Impact path and weight analysis

Place perception influenced health perception through multiple pathways. The three sub-dimensions showed clear hierarchical differences in effect estimates (Table 6). The association between landscape visual characteristics and physical comfort had the strongest standardized coefficient (*β* = 0.486, contribution rate 29.3%). Vegetation visibility and spatial openness substantially enhanced visitors’ evaluations of air quality and overall environmental comfort. Place emotional cognition ranked second in its influence on psychological restorativeness (*β* = 0.437, contribution rate 26.3%). Place identity and emotional attachment facilitated stress relief and attention restoration through psychological connections. Carbon-neutral characteristic perception retained an independently significant positive effect on health perception (*β* = 0.312, contribution rate 18.8%). Together, the three sub-dimensions formed a parallel structure – visual quality, emotional connection, and carbon-neutral cognition – through which place perception was associated with health perception.

Effect decomposition showed that the total effect of place perception on the health perception composite index was 1.235, with direct effects accounting for 68.4% and indirect effects via environmental behavior accounting for 31.6% (Table 6). The overall model fit (*R*^2^ = 0.617) indicated that the questionnaire-based place perception and environmental behavior variables explained a substantial proportion of variance in health perception.

Cross-park comparative analysis further revealed a gradient distribution of carbon-neutral characteristic perception path coefficients that closely matched each park’s carbon-neutral features (Table 9). Wenyu River Park, which has a complete carbon credit closed-loop system, recorded the highest carbon-neutral characteristic perception score (4.12) and the strongest path coefficient on health perception (*β* = 0.368). Urban Green Heart Forest Park showed intermediate scores (3.74) and a path coefficient of *β* = 0.298. Haotian Park, where carbon-neutral characteristics were least perceptible to visitors, recorded the lowest score (3.31) and the smallest path coefficient (*β* = 0.241). This gradient pattern further supports the existence of an independent net contribution from carbon-neutral-specific mechanisms to health perception, providing cross-case empirical evidence for separating carbon-neutral-specific effects from general green space effects.

**Table 9:** Comparison of independent path coefficients of carbon-neutral characteristic perception.

Park name	Carbon-neutral characteristic perception mean score	Carbon-neutral characteristic perception → Health perception path coefficient *β*	Significance level	Carbon-neutral characteristic configuration
Wenyu River Park	4.12	0.368***	*P* < 0.001	Complete carbon credit closed-loop system
Urban Green Heart Forest Park	3.74	0.298**	*P* < 0.01	Carbon neutrality experience route
Haotian Park	3.31	0.241*	*P* < 0.05	Construction-phase low-carbon technologies
Overall Mean	3.72	0.312***	*P* < 0.001	–

***p < 0.001, **p < 0.01, *p < 0.05; path coefficients are estimated independently while simultaneously controlling for landscape visual characteristics and place emotional cognition.

### 5.2 Association between pro-environmental behavior and health perception

#### 5.2.1 Health benefits of low-carbon behavior

Low-carbon behavior was positively correlated with health perception scores across all three parks. The underlying mechanisms appeared to involve behavioral guidance and self-efficacy activation specific to carbon-neutral parks, rather than merely increased physical activity.

The carbon credit system provides immediate, quantifiable feedback. When visitors complete specific low-carbon actions, they perceive their actual environmental contributions in real time. This feedback may help overcome the inherent spatiotemporal delay of low-carbon behavior effects, transforming abstract contributions into tangible outcomes and thereby enhancing perceived control. Path analysis confirmed that low-carbon behavior composite scores significantly affected both psychological restorativeness and physical comfort in all three parks (Table 10).

**Table 10:** Comparison of impact effects of low-carbon behavior participation on health perception.

Park name	Low-carbon behavior composite score	Impact on physiological comfort	Impact on psychological restorativeness	Impact on health perception composite index	High-frequency group health perception score	Low-frequency group health perception score	Inter-group difference
Wenyu River Park	3.68	0.289**	0.378***	0.336***	4.41	3.12	1.29***
Green Heart Park	3.47	0.267**	0.342***	0.308***	4.26	3.03	1.23***
Haotian Park	3.39	0.251*	0.318**	0.287**	4.18	2.97	1.21***
Overall Mean	3.51	0.269**	0.346***	0.310***	4.28	3.04	1.24***

***p < 0.001, **p < 0.01, *p < 0.05.

Participation in low-carbon behavior varied across parks. Wenyu River Park had the highest composite score (3.68) and the strongest health benefits, with the largest impacts on psychological restorativeness (*β* = 0.378) and physical comfort (*β* = 0.289), and the biggest inter-group difference in health perception between high- and low-frequency participants (1.29). Haotian Park recorded the lowest composite score (3.39) and more modest coefficients, while Urban Green Heart Forest Park occupied an intermediate position (3.47). The overall mean inter-group difference of 1.24 confirmed that higher participation frequency in low-carbon behavior was consistently associated with substantially elevated health perception scores across all three parks, demonstrating the substantive role of behavioral guidance in health promotion.

The positive association between low-carbon behavior and health perception can be further understood through Attention Restoration Theory (ART) and self-efficacy theory. The carbon credit system’s real-time, quantifiable feedback transforms abstract low-carbon actions into subjectively meaningful contributions. This immediate feedback acts as a source of “soft fascination” – a core ART component – capturing involuntary attention without cognitive overload. In addition, perceiving one’s own contribution to carbon reduction enhances visitors’ self-efficacy, which in turn is positively associated with psychological restoration. The significantly higher health perception scores among carbon credit users (composite score 4.28 vs. 3.04 for non-users, Table 10) are consistent with this dual-mechanism interpretation.

Taken together, the effect of low-carbon behavior on health perception is not a single linear pathway but rather the synergistic interplay of behavioral guidance (unique to carbon-neutral parks), self-efficacy activation, and the health benefits of physical activity.

#### 5.2.2 Psychological restorative effects of ecological experience

Ecological experience was strongly associated with psychological restoration scores. Natural contact and sensory immersion appeared to be underlying mechanisms, consistent with attention restoration theory. Behaviors such as observing plants and animals and participating in nature education activities may reduce cognitive load, lower perceived stress, and support emotional regulation. Ecological experience behaviors–observing plants, birds, and seasonal changes – also served as indicators of perceived biodiversity. Deeper engagement in biodiversity-rich areas was associated with significantly higher psychological restoration scores.

Path analysis showed that the impact coefficient of ecological experience on psychological restorativeness was 0.459 (*p* < 0.001), much higher than its coefficient on physical comfort (0.212, *p* < 0.05). Among the three parks, Wenyu River Park had the highest rate of deep participation in ecological experience and the most significant psychological recovery effect, owing to its rich biodiversity and well-developed nature education area. Visitors with high participation depth reported a psychological restoration score of 4.55, compared to 3.08 for those with shallow participation (mean difference = 1.47, *p* < 0.001). This pattern suggests that carbon-neutral parks, which often integrate functional biodiversity (e.g., carbon-sink plants, wetland habitats), provide dual benefits–carbon sequestration and psychological restoration–a synergy less commonly emphasized in conventional urban green spaces (Table 11).

**Table 11:** Comparison of impact effects of ecological experience participation depth on psychological restorativeness.

Park name	Ecological experience composite score	Psychological restorativeness composite score	Impact path coefficient on psychological restorativeness	Impact path coefficient on physiological comfort	Deep participation group psychological recovery score	Shallow participation group psychological recovery Score	Inter-group difference
Haotian Park	3.53	3.91	0.458***	0.213*	4.55	3.08	1.47***
Wenyu River Park	3.76	4.12	0.487***	0.226*	4.68	3.15	1.53***
Green Heart Park	3.38	3.74	0.432***	0.198*	4.42	3.01	1.41***
Overall Mean	3.56	3.92	0.459***	0.212*	4.55	3.08	1.47***

***p < 0.001, **p < 0.01, *p < 0.05.

#### 5.2.3 Comparative analysis of behavioral type differences

Different types of environmental behavior differed significantly in their effects on health perception. Using variance analysis and multiple comparison tests, we established the order of association strength. Ecological experience showed the strongest relationship with psychological restoration (*β* = 0.458, *p* < 0.001). Natural environment contact and sensory experience may directly trigger the attention restoration process. Low-carbon behavior correlated with the total health perception score at 0.308 (*p* < 0.001), a benefit mainly attributable to improved self-efficacy. Pro-environmental practices had the most prominent effect on environmental satisfaction (*β* = 0.392, *p* < 0.001), with volunteer activities helping to enhance place belonging. Paired t-tests showed that the psychological restoration score of ecological experience participants was 0.83 points higher than that of the low-carbon behavior group (*t* = 4.67, *p* < 0.001) and 1.05 points higher than the pro-environmental practices group (*t* = 5.42, *p* < 0.001). Visitors who engaged in all three behavior types had a health perception score of 4.38, which was 1.26 points higher than that of single-behavior participants. This indicates a synergistic enhancement effect of behavioral diversity.

### 5.3 Interaction patterns and mediation pathways

#### 5.3.1 Association between place perception and pro-environmental behavior

The association between place perception and pro-environmental behavior followed a “cognition-emotion-behavior” pattern, suggesting a potential transmission process. Landscape visual characteristics, as sensory stimulation sources, created comfortable visual environments through high green view index and good color harmony. This stimulated visitors’ initiative to explore and interact, thereby promoting ecological experience behaviors (*β* = 0.394, *p* < 0.001). Place identity acted as a value resonance mechanism. When visitors identified with the park’s carbon-neutral concept, their willingness to participate in pro-environmental practices increased significantly (*β* = 0.427, *p* < 0.001). Place attachment established a sense of belonging through emotional bonds, strengthening revisit intentions and behavioral engagement. For each unit increase in attachment, low-carbon behavior participation frequency rose by 0.356 units. Bootstrap mediation testing showed that place emotional cognition partially mediated the relationship between landscape visual characteristics and environmental behavior (indirect effect = 0.187, 95% CI = [0.094, 0.302]). This supports a mediation pathway of “high-quality place environment → place emotional identification → environmental behavior participation”, providing theoretical support for guiding visitor behavior through place creation.

#### 5.3.2 Mediation role of pro-environmental behavior

Research hypothesis H6 proposed that pro-environmental behavior mediates the relationship between place perception and health perception. Bootstrap mediation testing confirmed this hypothesis. As shown in Table 12, the total mediation effect was 0.186, accounting for 23.4% of the total effect (0.794). The 95% bias-corrected confidence interval [0.124, 0.256] excluded zero, supporting H6. Place perception not only directly promoted health perception (direct effect = 0.608, 76.6% of total) but also strengthened perceived health benefits indirectly by stimulating environmental behaviors – low-carbon behavior, ecological experience, and pro-environmental practices. This forms a transmission mechanism of “place cognition → behavioral participation → health benefits.”

**Table 12:** Mediation effect decomposition of pro-environmental behavior.

Mediation path	Direct effect	Indirect effect	Total effect	Mediation effect ratio	95% confidence interval	Significance
Place perception → Pro-environmental behavior → Health perception	0.608***	0.186***	0.794	23.4%	[0.124, 0.256]	Significant
Place perception → Health perception (direct effect)	0.608***	–	–	76.6%	[0.518, 0.698]	Significant
Pro-environmental behavior total mediation effect	–	0.186***	–	23.4%	[0.124, 0.256]	Significant

***p < 0.001, **p < 0.01, *p < 0.05.

Cross-park comparison revealed systematic differences in mediation effect strength across the three parks (Table 13). Wenyu River Park recorded the highest total mediation effect (0.284) and proportion (34.2%), attributable to its complete carbon credit system driving the strongest low-carbon behavior mediation effect (0.156). Urban Green Heart Forest Park recorded the lowest total mediation effect (0.216) and proportion (28.6%). Haotian Park occupied an intermediate position, with a total mediation effect of 0.243 and proportion of 31.7%. Across all three parks, ecological experience contributed meaningfully alongside low-carbon behavior. The overall mean mediation effect was 0.108 for ecological experience and 0.139 for low-carbon behavior, jointly accounting for the 31.5% overall mediation proportion. The consistency of significant mediation effects across parks, together with the gradient variation in mediation strength that matched each park’s carbon-neutral characteristics, provides robust empirical support for H6. It also demonstrates that the strength of behavioral mediation is shaped by the specific carbon-neutral features of individual parks.

**Table 13:** Decomposition of mediation effects of low-carbon behaviors and ecological experience.

Park name	Low-carbon behaviors mediation effect	Ecological experience mediation effect	Total mediation effect	Mediation effect proportion	Pure direct effect	95% confidence interval	Significance
Wenyu River Park	0.156**	0.128**	0.284***	34.2%	0.546***	[0.158, 0.421]	Significant
Green Heart Park	0.124*	0.092*	0.216*	28.6%	0.507***	[0.112, 0.338]	Significant
Haotian Park	0.138**	0.105*	0.243**	31.7%	0.524***	[0.134, 0.372]	Significant
Overall Mean	0.139**	0.108*	0.248**	31.5%	0.526***	[0.135, 0.377]	Significant

***p < 0.001, **p < 0.01, *p < 0.05.

#### 5.3.3 Moderation effects of demographic variables

Research Hypothesis H7 proposed that demographic characteristics significantly moderate the place perception–environmental behavior–health perception pathway. Using the merged sample of 420 valid responses across the three parks, PROCESS macro moderation analysis confirmed this hypothesis across multiple pathways (Table 14). Age significantly moderated the relationship between place perception and health perception (*β* = 0.163, *p* < 0.05). The path coefficient for middle-aged and older groups (*β* = 0.641) substantially exceeded that for younger groups (*β* = 0.478). Education level significantly moderated the effect of environmental behavior on health perception (*β* = 0.191, *p* < 0.01). Highly educated groups obtained more pronounced health benefits through behavioral participation (*β* = 0.589 vs. *β* = 0.398). Visit frequency strengthened the promoting effect of place attachment on health perception (*β* = 0.227, *p* < 0.01). High-frequency visitors showed markedly stronger path coefficients (*β* = 0.563 vs. *Β* = 0.336). Gender did not significantly moderate the ecological experience–psychological restorativeness pathway (*β* = 0.091, not significant). Thus, H7 was partially supported.

**Table 14:** Moderation effects of demographic variables on path coefficients.

Moderating variable	Moderation path	Interaction effect	Low-level group path coefficient	High-level group path coefficient	Inter-group difference	Significance level
Age	Place perception → Health perception	0.163*	0.478**	0.641***	0.163	*P* < 0.05
Education Level	Environmental behavior → Health perception	0.191**	0.398**	0.589***	0.191	*P* < 0.01
Visit Frequency	Place attachment → Health perception	0.227**	0.336*	0.563***	0.227	*P* < 0.01
Gender	Ecological experience → Psychological restorativeness	0.091	0.441**	0.482**	0.041	Not significant
***p < 0.001, **p < 0.01, *p < 0.05; Low-level group and high-level group are divided by median.						

### 5.4 Independent effects of carbon-neutral characteristic perception and group comparison

As shown in Table 15, carbon-neutral characteristic perception was positively correlated with health perception within the SEM model (*β* = 0.312, *p* < 0.001), controlling for landscape visual characteristics and place emotional cognition. Cross-park comparative analysis further revealed that path coefficients followed a gradient distribution consistent with each park’s carbon-neutral configuration: Wenyu River Park (*β* = 0.368), Urban Green Heart Forest Park (*β* = 0.298), and Haotian Park (*β* = 0.241). This confirms that the independent health promotion effect of carbon-neutral characteristic perception intensifies systematically with the richness of carbon-neutral infrastructure, thereby supporting H3.

**Table 15:** Independent effects of carbon-neutral characteristic perception and group comparison based on carbon credit system usage.

Analysis dimension	Indicator	Value	Significance level
Carbon-neutral characteristic perception path coefficient	*β* value	0.312	*P* < 0.001
Independent contribution rate	Variance explanation proportion	18.8%	–
Landscape visual characteristics path coefficient (reference)	*β* value	0.486	*P* < 0.001
Place emotional cognition path coefficient (reference)	*β* value	0.437	*P* < 0.001
Usage group health perception index mean (*n* = 213)	Composite score	4.24	–
Non-usage group health perception index mean (*n* = 189)	Composite score	3.61	–
Inter-group difference	Mean difference	0.63	*P* < 0.001
Usage group environmental behavior → Health perception path coefficient	*β* value	0.491	*P* < 0.001
Non-usage group environmental behavior → Health perception path coefficient	*β* value	0.298	*P* < 0.01
Bootstrap 95% confidence interval	Inter-group difference interval	[0.097, 0.268]	Significant

Group comparison analysis provided further validation (Table 15). The usage group – primarily Wenyu River Park visitors who engaged with the carbon credit system, supplemented by Urban Green Heart Forest Park visitors who participated in the carbon neutrality experience route (*n* = 213)–recorded a significantly higher environmental behavior → health perception path coefficient (*β* = 0.491, *p* < 0.001) than the non-usage group (*n* = 189, *β* = 0.298, *p* < 0.01). The Bootstrap 95% confidence interval [0.097, 0.268] excluded zero. The usage group’s mean health perception score (4.24) exceeded that of the non-usage group (3.61) by 0.63 points (*p* < 0.001). The convergence of evidence across the overall model, cross-park gradient comparison, and group comparison collectively confirms that the carbon credit behavioral guidance mechanism is associated with higher health perception scores via self-efficacy activation, independent of conventional urban green space benefits.

### 5.5 Discussion

The findings of this investigation provide empirical validation for the theoretical framework that links place perception, pro-environmental behavior, and health perception in carbon-neutral theme parks. Beyond replicating findings derived from conventional urban green space research, our analysis identifies three theoretical extensions that are distinctive to the carbon-neutral context.

First, this study extends Attention Restoration Theory (ART) to carbon-themed park environments. Traditional ART emphasizes the restorative value of natural landscapes. By contrast, our findings suggest that human-designed, carbon-focused environments—particularly the visibility of low-carbon facilities, real-time carbon credit feedback, and cognitive engagement with carbon-sink plant displays—can likewise trigger attention restoration and facilitate psychological recovery. The independent positive association between carbon-neutral characteristic perception and health perception (*β* = 0.312, *p* < 0.001, Table 15), after adjusting for landscape visual characteristics and place emotional cognition, indicates that these novel environmental features serve as restorative resources that cannot be reduced solely to general greenery or place attachment.

Second, we propose a “behavioral feedback–value identification–health perception” pathway. Whereas traditional environmental behavior theories primarily emphasize the health benefits of the behavior itself (e.g., physical activity), our multi-group comparison (Table 14) reveals that the path coefficient from environmental behavior to health perception is markedly higher among carbon credit users (*β* = 0.487) than among non-users (*β* = 0.298). This finding indicates that real-time, quantifiable feedback from the carbon credit system reinforces the behavior–health perception linkage by enhancing self-efficacy and perceived contribution — a digitally augmented mechanism previously undocumented in ordinary green space contexts.

Third, we integrate “carbon mission identity” into the framework of place attachment. Place attachment within carbon-neutral parks appears to incorporate an additional “global–local” dimension. Specifically, visitors’ identification with the park’s carbon-neutral mission bridges local environmental experience to broader global climate governance, thereby potentially amplify psychological benefits beyond those derived from place attachment in conventional urban parks. This is empirically supported by the carbon-neutral characteristic perception sub-dimension item “perceived differentiation of low-carbon theme” (Table 2) and its associated significant path coefficient (*β* = 0.312).

Taken together, these theoretical extensions imply that carbon-neutral theme parks are not simply well-designed urban green spaces; rather, they generate distinct cognitive, behavioral, and health-related pathways that deserve separate theoretical attention.

Carbon-neutral theme parks typically contain two conceptually distinct types of natural features: rewilding elements and display-oriented carbon-sink plantings. Rewilding may facilitate psychological restoration through unpredictable fascination and perceived ecological connectedness, whereas carbon-sink displays are likely to operate via cognitive mastery and goal-consistent feedback regarding climate action. This study could not fully separate these pathways, as the three case parks intermingle both features. Future research adopting experimental designs or fine-grained photo-sorting tasks is required to examine whether rewilding and functional plantings engage distinct mediators, which would inform more nuanced health-oriented park planning.

## 6 Conclusion

With three carbon-neutral theme parks in Beijing as a comparative case portfolio, this study integrates carbon-neutral-specific variables into the existing place perception → environmental behavior → health perception framework. It identifies three potential pathways that extend prior frameworks. The “technology visibility–behavioral feedback” pathway suggests a potential self-efficacy-related enhancement associated with real-time feedback from low-carbon technologies, as evidenced by higher health perception scores among carbon credit system users. The “cognitive reconstruction — meaning attribution” pathway integrates carbon-neutral knowledge acquisition into ecological experience activities, thus broadening the emotional dimension embedded in place attachment theory. The “system coupling — multi-scale association” pathway shifts traditional place perception from a local focus to the level of global environmental governance, explicitly incorporating carbon-neutral mission identity. Collectively, these extensions furnish a theoretical basis for health-related research on carbon-neutral urban public spaces that departs meaningfully from general green space planning. In addition, park managers should deploy age-targeted behavioral interventions. For middle-aged and older visitors — who show notably stronger positive links between place perception and health perception (Table 14) — enhancing narrative and interpretive signage concerning carbon benefits would be effective. For younger visitors, gamified features within the carbon credit system (e.g., point challenges, real-time feedback) can enhance initial engagement and facilitate habit formation.

The study has three limitations. First, although a three-park comparative framework is adopted, the sample size of 420 valid responses and the geographic restriction to Beijing restrict the statistical generalizability of our findings. Future research could broaden the sample scope and incorporate conventional urban parks from diverse regional contexts to better delineate the boundary conditions of the identified extension pathways. Second, health perception relies primarily on self-reported data. Subsequent research could incorporate objective physiological indicators, such as heart rate variability and cortisol levels, to strengthen causal inference. Third, due to the absence of a traditional urban park control group, the net contribution of carbon-neutral-specific effects relative to general green space effects has not been completely separated. Future research could achieve more rigorous effect separation by incorporating conventional park control groups or employing counterfactual scenario designs. Additionally, the mechanisms through which emerging smart technologies, such as augmented reality and the Internet of Things, influence on visitor-environment interaction and health perception represent worthwhile directions for in-depth exploration.

## Data Availability

The raw data supporting the conclusions of this article will be made available by the authors, without undue reservation.
